# Congenital Colour Vision Deficiency among Patients Attending Outpatient Department of Ophthalmology in a Tertiary Care Centre: A Descriptive Cross-sectional Study

**DOI:** 10.31729/jnma.7319

**Published:** 2022-03-31

**Authors:** Priyanka Shrestha, Pranil Man Singh Pradhan

**Affiliations:** 1 Department of Ophthalmology, Kathmandu Medical College and Teaching Hospital, Kathmandu, Nepal; 2 Department of Community Medicine, Maharajgunj Medical Campus, Institute of Medicine, Tribhuvan University, Kathmandu, Nepal

**Keywords:** *colour vision defect*, *Nepal*, *prevalence*

## Abstract

**Introduction:**

Congenital colour vision deficiency may affect a person's day-to-day activity and may also affect the choice of occupation a person chooses. This study aims to find the prevalence of congenital colour vision defects in patients presenting in outpatient department of Ophthalmology in a tertiary care centre.

**Methods:**

A descriptive cross-sectional study was conducted in a tertiary care centre after receiving ethical clearance from the Institutional Review Board of Kathmandu Medical College and Teaching Hospital (Reference number: 1006202102). The study was conducted for a 3 months period from 2021 July to 2021 September. Research participants were selected by the convenience sampling technique. A detailed ophthalmological examination was performed and colour vision was tested using Ishihara pseudoisochromatic colour vision chart. Only congenital colour vision defects were included in the study. Statistical Package for the Social Sciences version 20.0 was used for data analysis. Point estimate at 95% confidence interval was calculated along with frequency and proportion for binary data.

**Results:**

The overall prevalence of congenital colour vision deficiency was 14 (5.24%) (2.54-7.86 at 95% Confidence Interval). The prevalence of congenital colour vision defects in females was 1 (0.74%) and in males was 13 (9.77%). The mean age of the participants with congenital colour vision deficits was 27.42±7.90 years.

**Conclusions:**

The prevalence of congenital colour vision deficiency was similar to the prevalence in other studies done in a similar setting. Awareness should be raised about this condition and people need to be screened at an early age to prevent disappointments in career choices later in life.

## INTRODUCTION

Colour vision deficiency can be congenital or acquired. Congenital colour vision deficiency is mostly an X-linked recessive disorder, therefore, are more commonly found in males and incidence varies from race to race.^12^ Congenital colour vision deficiency causes difficulties in differentiating colours in the red/green spectrum and are bilateral and nonprogressive.^3,4^ Acquired colour vision defects may be due to other ocular and intracranial causes, diabetic retinopathy, drugs, ageing, macular degeneration, hypertension, and glaucoma.

The prevalence of congenital colour vision deficiency in the general population is 8% in males and 0.5% in females.^5^

Severe colour vision deficiency can significantly affect a person's life and limit them from various professions like driving, military, pilots, air traffic controllers and health services.^6^ So it is important to know that congenital colour vision defects occur and if severe may affect the person in day-to-day life or in joining various professions.

Therefore, this study aimed to study the prevalence of congenital colour vision defects in a tertiary care centre.

## METHODS

A descriptive cross-sectional study was conducted among 267 participants in the Department of Ophthalmology, Kathmandu Medical College and Teaching Hospital during a period of 3 months from July 2021 to September 2021. All the research participants underwent detailed ocular examinations. Participants with history of diabetes, glaucoma, macular and retinal diseases, cataract or any other media opacity, red eye, optic nerve disease, Alzeimers disease, Parkinson's disease, Multiple sclerosis, chronic alcoholism, leukaemia, sickle cell disease, subjects with intellectual disability not able to read the chart properly, subjects under various medications which can affect colour vision and subjects with any other conditions affecting colour vision were excluded from the study. Ethical clearance was taken from the Institutional Review Board of Kathmandu Medical College Teaching Hospital (Reference number: 1006202102) and researchers have adhered to the tenets of the Declaration of Helsinki. Convenience sampling method was used. The sample size was calculated using the following formula:

n = (Z^2^ × p × q) / e^2^

  = (1.96^2^ × 0.17 × 0.83) / 0.05^2^

  = 217

Where,

n = minimum required sample size
Z = 1.96 at 95% Confidence Interval (CI)
p = prevalence from a similar study, 17.8%^6^
q = 1-p
e = margin of error, 5%

With a 10% non-response rate, the sample size was calculated to be 239. A total of 267 participants were included in the study.

All patients between the age group of 10-40 years fulfilling the inclusion criteria were included in the study. Written consent was taken from all the participants. For participants less than 18 years, written consent from parents was taken. Detailed systemic and ocular history was taken and a complete ocular examination was done. Distant visual acuity was taken using a self-illuminated Snellen's chart at a 6-metre distance. Slit-lamp biomicroscopy was done to evaluate the anterior and posterior segments and patients with a normal anterior and posterior segment were included in the study. Colour vision was tested with an Ishihara pseudoisochromatic (38-plate edition). The test was performed under normal daylight illumination. Numerals or path tracings were used to detect colour vision defects. A pseudoisochromatic plate is composed of coloured dots in a background of differently coloured dots. These plates are chosen such that an X-linked colour blind patient is not able to see the figure which is easily seen by a normal person. Congenital colour vision defects are protan and deutan colour vision defects and these plates are effective in detecting 9095% of the congenitally colour defective patients.^7^ There are screening plates and diagnostic plates in the chart. The screening plates are the vanishing plates, transformation plates and the hidden plates. Diagnostic plates differentiate deutans from protans. In the 38 plates edition, the plates 1 to 21 are screening plates and plates 22 to 25 differentiate protans and deutans. Four or less errors are considered normal, eight or more errors are considered deficient.^8^ After the colour vision test, few questions were asked regarding the awareness of colour vision defects and the various professions that require prior colour vision testing and a normal colour vision before joining. Statistical Package for the Social Sciences version 20.0 was used for data analysis. Point estimate at 95% confidence interval was calculated along with frequency and proportion for binary data.

## RESULTS

A total of 267 research participants were enrolled in the study, the prevalence of congenital colour vision defects was 14 (5.24%) (2.54-7.86 at 95% Confidence Interval). The prevalence of congenital colour vision defects in females was 1 (0.74%) and in males was 13 (9.77%). The mean age of the participants with congenital colour vision deficits was 27.42±7.90 years (Table 1).

**Table 1:** Distribution of patients with congenital colour vision defects according to age (n= 14).

Range of age (in years)	n (%)
10-20	3 (21.42)
21-30	5 (35.71)
31-40	6 (42.86)

Based on ethnicity, the maximum number of subjects who were congenital colour vision deficient were Brahmins, 6 (42.90%) (Table 2).

**Table 2:** Distribution of congenital defects according to ethnicity (n= 14)

Ethnicity	n (%)
Brahmins	6 (42.85)
Magars	3 (21.42)
Newars	2(14.28)
Other Janajatis	2 (14.28)
Others	1 (7.14)

Among the patients who had congenital colour vision defects, 8 were red-green colour blind (4 each in the strong deutan and strong protan group) (Table 3).

**Table 3:** Distribution according to the type of congenital colour vision defects (n= 14).

Type	n (%)
Strong deutan	4 (28.57)
Strong protan	4 (28.57)
Total colour blindness	6 (42.85)

Among the 14 patients who had congenital colour vision deficits, 8 (57.1%) were not aware of their condition and the remaining 6 (42.9%) were aware of their condition. Nine (64.3%) did not have any difficulty differentiating colours, 4 (28.6%) had difficulty differentiating colours and 1 (7.1%) did not give any response. The patients with congenital colour vision defects were asked whether they knew the need for normal colour vision in certain occupations. Nine (64.3%) had an idea regarding the need for a normal colour vision for certain occupations, the rest 5 (35.7%) did not have any knowledge. Among the positive responses, 9 (64.28%) mentioned driving licence and 1 (7.14%) mentioned pilots among the occupations requiring a normal colour vision.

## DISCUSSION

Colour vision defects can be congenital or acquired. Most congenital colour vision defects are red-green deficiencies and are X-linked recessive traits. Therefore, they occur predominantly in males but are transmitted by females who are mostly carriers.^9^ Acquired colour vision defects are mostly secondary to diseases of the retina or the optic nerve.

The prevalence of congenital colour blindness varies in different geographical areas and differs from race to race.^10^ The overall prevalence of congenital colour vision defects in our study was 5.2% which was comparable to the prevalence of congenital colour vision defects in another study done in Nepal.^11^ The prevalence of congenital colour vision deficiency was higher in the male population 9.8% which was similar to the prevalence of congenital colour vision deficiency in studies done in the Nepalese male population.^5,9^ The prevalence of congenital colour vision deficiency in females in our study was 0.7% which was slightly lower than the prevalence of congenital colour vision deficiency in females in a study done in the western region of Nepal.^5^ In comparison to other countries the prevalence of congenital colour vision defects in our study was higher than that done in Malaysia (3.2%),^10^ South Africa (2.2%),^12^ Southern Ethiopia (4.1%),^13^ Southwest Nigeria (2.3%),^14^ and India (2.98%).^15^ Since congenital colour vision defect is an X-linked disease its frequency is higher in males than in females. In our study too, it was more prevalent in males than in females. In this study, based on ethnicity, congenital colour vision defect was more common in Brahmins followed by Magar populations. In a study done by Godar S, el al. it was more common in Chettris followed by Brahmins.^5^ In our study, only congenital colour vision defects were included which could have led to differences in results. In another study done by Malhotra NB, et al. congenital colour vision deficiency was more common in Brahmins which was similar to the results of our study.^16^ However, since the sample size of our study was small, the ethnic distribution of congenital colour vision deficiency would not be the exact representation of the Nepalese population. The red-green deficiency was more common than total colour blindness in our study. The results of which are similar to other studies.^5,17^ However, The occurrence of deutan and protan deficiency was the same in our studies unlike in other studies where deutan defects were more common than protan defects,^13,12,18^ and similar to the finding of a study done by Ugalahi M, et al. where the deutan to protan ratio was 1:1.1.^14^

In our study, 57.1% who had congenital colour vision defects were unaware of their condition. Among them 64.3% were aware of the need for intact colour vision for certain professions. Normal colour vision is a must for certain professions. Aviation, defence, healthcare and medicine, painters, decorators, interior designers, hairdressers and drivers all require normal colour vision.^1,6^ People with congenital colour vision defects need to know about their condition early to prevent disappointments later in life due to job restrictions in many fields which require a normal colour vision. Congenital colour vision defect is a genetic disease. Even though no definite treatment is available, its identification early in life may help in premarital or preconception genetic counselling as well.^18^ Among the positive respondents who were aware of the need for an intact colour vision for various occupations, 64.28% mentioned driving licence followed by pilots (7.14%). Even though 64.3% knew that an intact colour vision is required for certain professions the knowledge was limited to only driving licence and pilots. This showed that they were unaware of the many other occupations that required intact colour vision.

Due to the small sample size in this study, the prevalence of congenital colour blindness may not be the exact representation of the Nepalese population. Our study used Ishihara pseudoisochromatic charts for detection of colour blindness which only detects red-green deficiency. It would have been better to detect colour blindness with an anomaloscope that would qualitatively and quantitatively classify colour blindness. However, due to the unavailability of an anomaloscope, we were unable to use it in our study.

## CONCLUSIONS

The prevalence of congenital colour vision deficiency was similar to the prevalence in other studies done in a similar setting. The prevalence was higher in males than in females which was similar to findings of studies done in similar settings. Even though a majority of subjects with congenital colour vision deficiency were aware of the need of intact colour vision for certain occupations the knowledge of specific occupations requiring normal colour vision were limited among them. Therefore, it is important to screen people for colour vision at an earlier age to prevent disappointments later in life while choosing various occupations and also to raise awareness among the population.
